# Fundamental Activity Constraints Lead to Specific Interpretations of the Connectome

**DOI:** 10.1371/journal.pcbi.1005179

**Published:** 2017-02-01

**Authors:** Jannis Schuecker, Maximilian Schmidt, Sacha J. van Albada, Markus Diesmann, Moritz Helias

**Affiliations:** 1 Institute of Neuroscience and Medicine (INM-6) and Institute for Advanced Simulation (IAS-6) and JARA BRAIN Institute I, Jülich Research Centre, Jülich, Germany; 2 Department of Psychiatry, Psychotherapy and Psychosomatics, Medical Faculty, RWTH Aachen University, Aachen, Germany; 3 Department of Physics, Faculty 1, RWTH Aachen University, Aachen, Germany; University College London, UNITED KINGDOM

## Abstract

The continuous integration of experimental data into coherent models of the brain is an increasing challenge of modern neuroscience. Such models provide a bridge between structure and activity, and identify the mechanisms giving rise to experimental observations. Nevertheless, structurally realistic network models of spiking neurons are necessarily underconstrained even if experimental data on brain connectivity are incorporated to the best of our knowledge. Guided by physiological observations, any model must therefore explore the parameter ranges within the uncertainty of the data. Based on simulation results alone, however, the mechanisms underlying stable and physiologically realistic activity often remain obscure. We here employ a mean-field reduction of the dynamics, which allows us to include activity constraints into the process of model construction. We shape the phase space of a multi-scale network model of the vision-related areas of macaque cortex by systematically refining its connectivity. Fundamental constraints on the activity, i.e., prohibiting quiescence and requiring global stability, prove sufficient to obtain realistic layer- and area-specific activity. Only small adaptations of the structure are required, showing that the network operates close to an instability. The procedure identifies components of the network critical to its collective dynamics and creates hypotheses for structural data and future experiments. The method can be applied to networks involving any neuron model with a known gain function.

## Author Summary

The connectome describes the wiring patterns of the neurons in the brain, which form the substrate guiding activity through the network. The influence of its constituents on the dynamics is a central topic in systems neuroscience. We here investigate the critical role of specific structural links between neuronal populations for the global stability of cortex and elucidate the relation between anatomical structure and experimentally observed activity. Our novel framework enables the evaluation of the rapidly growing body of connectivity data on the basis of fundamental constraints on brain activity and the combination of anatomical and physiological data to form a consistent picture of cortical networks.

## Introduction

The neural wiring diagram, the connectome, is gradually being filled through classical techniques combined with innovative quantitative analyses [1, 2] and new technologies [3, 4]. The connectivity between neurons is considered to shape resting-state and task-related collective activity [5, 6]. For simple networks, clear relationships with activity are known analytically, e.g., a dynamic balance between excitatory and inhibitory inputs in inhibition-dominated random networks leads to an asynchronous and irregular state [7–9]. Structures and mechanisms underlying large-scale interactions have been identified by means of dynamical models [10, 11] and graph theory [12, 13]. Furthermore, the impact of local network structure on spike-time correlations is known in some detail [14–16]. Under certain conditions, there is a one-to-one mapping between correlations in neuronal network activity and effective connectivity, a measure that depends on the network structure and on its activity [17]. In conclusion, anatomy does not uniquely determine dynamics, but dynamical observations help constrain the underlying anatomy. Despite advances in understanding simple networks, a complete picture of the relationship between structure and dynamics remains elusive.

**Fig 1:**
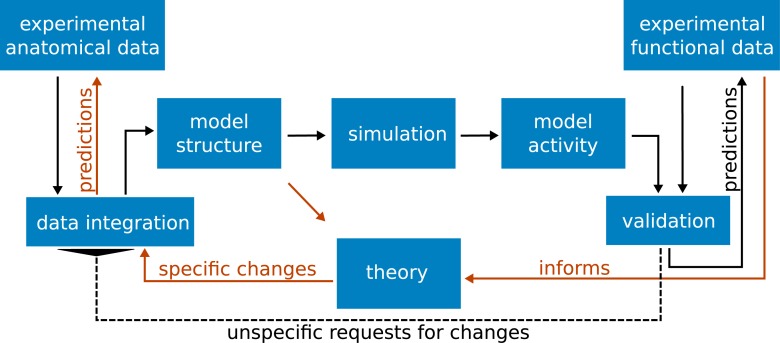
The integrative loop between experiment, model, and theory. Black arrows represent the classical forward modeling approach: Experimental anatomical data are integrated into a model structure, which gives rise to the activity via simulation. The model activity is compared with experimental functional data. The usual case of disagreement leads to the need to change the model definition. By experience and intuition the researcher identifies the parameters to be changed, proceeding in an iterative fashion. Once the model agrees well with a number of experimental findings, it may also generate predictions on the outcome of new experiments. Red arrows symbolize our new approach: informed by functional data, an analytical theory of the model identifies critical connections to be modified, refining the integration of data into a consistent model structure and generating predictions for anatomical studies.

Fig 1 visualizes the integrative loop between experiment, model, and theory that guides the investigation of structure and dynamics. In the traditional forward modeling approach, structural data from experimental studies determine the connectivity between model neurons. Combined with the specification of the single-neuron dynamics and synaptic interactions, simulations yield a certain network dynamics. There is a fundamental problem with this approach.

Despite the impressive experimental progress in determining network parameters, any neural network model is underdetermined, because of the sheer complexity of brain tissue and inevitable uncertainties in the data. For instance, counting synapses on a large scale presently takes a prohibitive amount of time, and no available technique allows determining precise synaptic weights for entire neural populations. Although it may be possible to quantify the full connectome of an individual in future, inter-individual variability will require modelers to express connectivity as connection probabilities or rules of self-organization if they want to learn about general principles of the brain. In modeling studies, parameters are usually tuned manually to achieve a satisfactory state of activity, which becomes unfeasible for high-dimensional models due to the size of the parameter space. In particular, it is a priori unclear how parameters of the model influence its activity. In consequence, modifications cannot be performed in a targeted fashion, and it is difficult to find a minimal set of modifications necessary for aligning a model with experimental activity data.

Overcoming this problem requires a shift of perspective. Instead of regarding the model exclusively in a forward manner, generating predicted activity from structure, we in addition consider the system in a reverse manner, predicting the structure necessary to explain the observed activity. Our theory, starting from a mean-field description, provides a direct link between structure and activity. In contrast to simulations, the theory is invertible, which we exploit to identify connections critical for the dynamics and to find a minimal set of modifications to the structure yielding a realistic set point of activity. The predicted alterations generate hypotheses on brain structure, thus feeding back to anatomical experiments.

Applying the method to a multi-scale network model of the vision-related areas of macaque cortex, we derive targeted modifications of a set of critical connections, bringing the model closer to experimental observations of cortical activity. Based on the global properties of the bistable phase space of the model, the method refines the model’s construction principles within experimental uncertainties and identifies the connections that decisively shape the dynamics. Preliminary results have been presented in abstract form [18].

## Results

### Global stability in a simple network

We consider networks with neurons structured into *N* interconnected populations. A neuron in population *i* receives *K*_*ij*_ incoming connections from neurons in population *j*, each with synaptic efficacy *J*_*ij*_. Additionally, each neuron is driven by *K*_ext_ Poisson sources with rate *ν*_ext_ and synaptic efficacy *J*_ext_. All neurons in one population have identical parameters, so that we can describe the network activity in terms of population-averaged firing rates *ν*_*i*_.

Our method is applicable if the employed neuron model has a known gain function, either analytically or as a function obtained by interpolating numerical results from simulations. In this study, we model single cells as leaky integrate-and-fire model (LIF) neurons (see “Methods”). The possible stationary states of these networks are characterized by the firing rates that are equilibria of

$$\dot{\nu }≔\frac{d\nu }{ds}=\Phi \left(\nu ,A\right)-\nu ,$$ (1)

where *s* is a pseudo-time. The gain function **Φ** is known analytically and ***A*** indicates its dependence on the model parameters {***K***, ***J***, *ν*_ext_, …} (see “Methods”).

**Fig 2:**
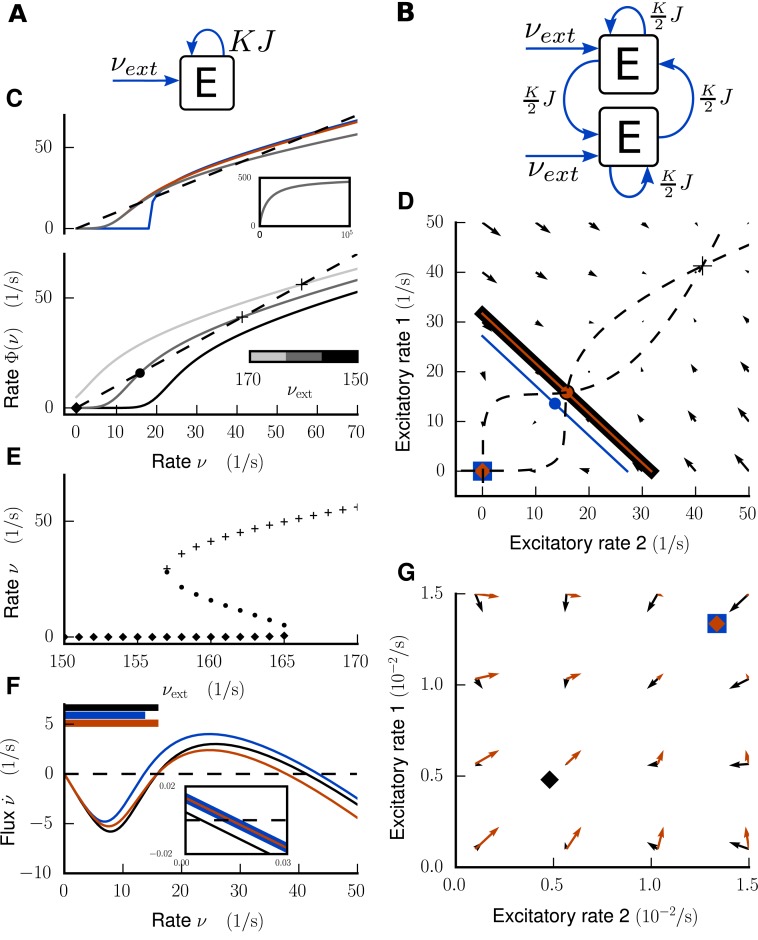
Activity flow in an illustrative network example. **Left column:** Global stability analysis in the single-population network. **A** Illustration of network architecture. **C** Upper panel: Input-output relationship $\Phi (\nu ,\nu_{\text{ext}})$ for external Poisson drive $\nu_{ext}=160\;\frac{1}{s}$ shown in gray. In addition $\Phi (\nu ,\nu_{\text{ext}})$ for $\tau_{r}=0$ for the noiseless case (blue) and the noisy case (red). The inset shows the gray curve over a larger input range. Lower panel: $\Phi (\nu ,\nu_{\text{ext}})$ for different rates of the external Poisson drive $\nu_{ext}=[150,160,170]\;\frac{1}{s}$ from black to light gray. Intersections with the identity line (dashed) mark fixed points of the system, which are shown in **E** as a function of *ν*_ext_. **F** Flux $\dot{\nu }$ in the bistable case for $\Phi (\nu_{ext}=160\frac{1}{s},\;K)$ in black, $\Phi (\nu_{ext}^{\prime }=161\frac{1}{s},\;K)$ in blue, and modified system $\Phi (\nu_{ext}^{\prime }=161\frac{1}{s},\;K^{\prime })$ in red. Intersections with zero (dashed line) mark fixed points. The inset shows an enlargement close to the LA fixed point. Horizontal bars at top of figure denote the size of the basin of attraction for each of the three settings. **Right column:** Global stability analysis in the network of two mutually coupled excitatory populations. **B** Illustration of network architecture. **D** Flow field and nullclines (dashed curves) for $\text{Φ}(\nu_{ext}=160\frac{1}{s},\;K)$ and separatrices (solid lines), LA fixed point (rectangle), HA fixed point (cross) and unstable fixed points (circles) for $\text{Φ}(\nu_{ext}=160\frac{1}{s},\;K)$ in black, $\text{Φ}(\nu_{ext}=161\frac{1}{s},\;K)$ in blue, and $\text{Φ}(\nu_{ext}=161\frac{1}{s},\;K^{\prime })$ in red. The red separatrix and the red unstable fixed point coincide with the black ones. **G** Enlargement of D close to the LA fixed points. Flow field of original system shown in black, of modified system in red.

The input-output relationship **Φ** typically features a non-linearity which, in combination with feedback connections, can cause multi-stability in the network. In particular excitatory-excitatory loops cause the system defined by Eq (1) to exhibit multi-stable behavior in the stationary firing rates. A necessary condition for the bistability is that the transfer function has an inflection point. The LIF neuron model can have such an inflection point, originating from the interplay of its leak term and the threshold. Less realistic neuron models, such as the perfect integrate-and-fire model, do not have such an inflection point. To illustrate its origin, we first consider the noiseless case [19] without absolute refractoriness (*τ*_r_ = 0). The transfer function initially grows from zero with infinite slope due to the threshold and crosses the identity line (Fig 2C). For larger input the leak term can be neglected and the transfer function approaches a linear function with finite slope $\frac{1}{\tau_{\text{m}}}\;\frac{R}{V_{\theta }-V_{r}}$ (see, e.g., [20], eq 11), equivalent to a perfect integrator. This is only possible with a negative curvature at intermediate rates, i.e., a reduction in the slope, which makes the transfer function cross the identity line another time, causing the bistability. Network-generated noise only affects the low-rate regime where it smears out the kink causing the transfer function to grow from zero with positive curvature (see, e.g. [21], eq. 22). Importantly, the qualitative picture, i.e., the bistable behavior, is not altered. A finite refractory period only has an effect for very high input values where the transfer function saturates at 1/*τ*_r_ = 500 spikes/s for the given parameters.

The basic problem is that there is a trade-off between excitation at the fixed points and their stability. In particular, exciting the model to bring a fixed point closer to experimental observations requires a method to preserve its stability. We achieve this by controlling the influence of the excitatory-excitatory loops on the phase space of the network.

As an illustration we first study the mechanism using the simple network architecture depicted in Fig 2A: a population of excitatory neurons is coupled to itself with indegree ***K*** and is driven by external Poisson sources with the same indegree *K*_ext_ = *K* and rate *ν*_ext_. All connections have identical synaptic weights *J*. An increase in the external drive shifts the input-output relationship **Φ**(*ν*, *ν*_ext_) of this one-dimensional system (Fig 2C) to the left. The bifurcation diagram is shown in Fig 2E: for low *ν*_ext_ there is only one fixed point with low activity (LA). When increasing *ν*_ext_, an additional pair of fixed points of which one is stable and the other is unstable emerges via a saddle-node bifurcation, leading to a bistable system. The second stable fixed point exhibits high firing rates, denoted as the high-activity (HA) state. For even higher values of *ν*_ext_, the LA state loses stability.

The equilibria, given by the zeros of the velocity $\dot{\nu }$ in the bistable case, are shown in Fig 2F. An increase of the drive on the one hand increases the firing rate of the LA fixed point (inset) but on the other hand reduces its basin of attraction, indicated by the colored bars in the top left corner. For illustrative purposes, we extend the problem to two dimensions by splitting the excitatory population into two subpopulations of equal size (Fig 2B), mimicking a loop between excitatory populations in the model of the vision-related areas of macaque cortex. The corresponding (symmetric) two-dimensional phase space is shown in Fig 2D. The basin of attraction for the LA fixed point, limited by the separatrix [22], is reduced with increasing external drive.

Since we have a bistable system, there must be at least one unstable fixed point on the separatrix at the intersection of the nullclines, i.e., the subspace for which the velocity ${\dot{\nu }}_{i}$ in direction *i* vanishes. We use the unstable fixed point to preserve the basin of attraction when the external drive *ν*_ext_ is increased. For this purpose, we modify the connectivity $K\to K^{\prime }$ to reverse the shift of the unstable fixed point due to the parameter change $\nu_{ext}\to \nu_{ext}^{\prime }$ (see “Methods” for a detailed derivation). Since the separatrix follows the unstable fixed point, this approximately restores the original basin of attraction.

The resulting velocity of the system $\Phi (\nu_{ext}^{\prime },K^{\prime })$ (Φ defines the system Eq (1)) is shown in Fig 2F. The firing rate in the LA fixed point is increased as desired (inset), and the unstable fixed point coincides with that obtained in the original system Φ(*ν*_ext_, *K*). This pattern of fixed points is also indicated by the zero vectors of the velocity field $\dot{\nu }$ (Fig 2D). The separatrix follows the unstable fixed point, and the basin of attraction in the system $\Phi (\nu_{ext}^{\prime },K^{\prime }$) is restored to that in the original system **Φ**(*ν*_ext_, *K*). Fig 2G shows the behavior of the LA fixed point in more detail. The modification of *K* does not noticeably change the location of the LA fixed point. In conclusion, the method allows us to increase the firing rates in the LA fixed point without modifying its basin of attraction.

The purely excitatory network is the simplest model to explain a phase space configuration with a LA and a HA fixed point. Inhibitory feedback is not necessary for this bistability, but it would certainly alter the input-output relationship. For example the classical excitatory-inhibitory network [21] in the balanced regime has an input-output relationship with a negative slope and thus only one fixed point exists. However, if a pair of such balanced E-I networks is coupled by sufficiently strong mutually excitatory connections, these connections cause a bistability in a similar manner. Thus the mechanism shown in the purely excitatory network can also lead to the emergence of a HA attractor in more complex networks with inhibition.

### Bistability in the multi-scale network model

**Fig 3:**
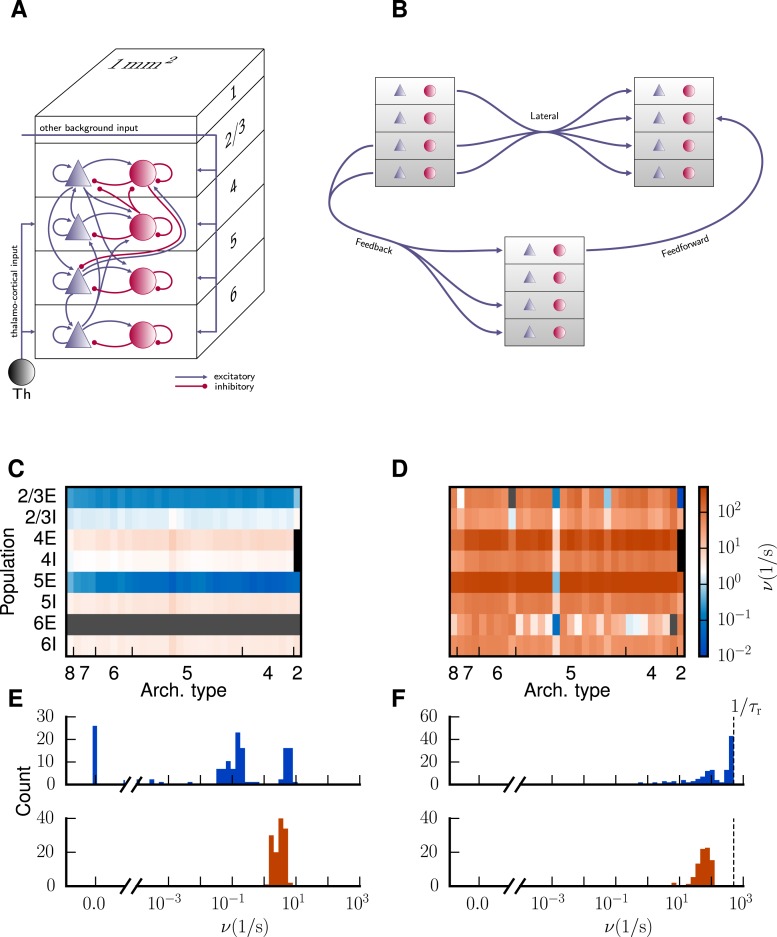
Bistability of the model. **A** Sketch of the microcircuit model serving as a prototype for the areas of the multi-area model (figure and legend adapted from figure 1 of Potjans and Diesmann [23], with permission). **B** Sketch of the most common laminar patterns of cortico-cortical connectivity of the multi-area model. **C** Population-averaged firing rates encoded in color for a spiking network simulation of the multi-area model with low external drive (κ=1.0). **D** As C but for increased external drive κ=1.125. The color bar refers to both panels. Areas are ordered according to their architectural type along the horizontal axis from V1 (type 8) to TH (type 2) and populations are stacked along the vertical axis. The two missing populations 4E and 4I of area TH are marked in black and firing rates $<10^{-2}\;\text{Hz}$ in gray. **E** Histogram of population-averaged firing rates shown in **C** for excitatory (blue) and inhibitory (red) populations. The horizontal axis is split into linear- (left) and log-scaled (right) ranges. **F** as E corresponding to state shown in D.

We investigate a multi-scale network model of cortical areas to understand the structural features essential for a realistic state of baseline activity. The model extends and adapts the microcircuit model presented in [23], which covers 1 mm^2^ surface area of early sensory cortex (Fig 3A), to all vision-related areas of macaque cortex (Fig 3B). Based on the microcircuit model, an area is composed of 4 layers (2/3, 4, 5, and 6) each having an excitatory (E) and an inhibitory (I) neural population, except parahippocampal area TH, which consists of only 3 layers (2/3, 5, and 6). A detailed description of the data integration is given in [24].

Simulations of the model (Fig 3C) reveal that, though realistic levels of activity can be achieved for populations in layers 2/3 and 4, populations 5E and 6E of the majority of areas show vanishingly low or zero activity in contrast to empirical data [25, 26]. Inputs from subcortical and non-visual cortical areas are modeled as Poissonian spike trains, whose rate *ν*_ext_ is a free, global parameter. To elevate the firing rates in the excitatory populations in layers 5 and 6, we enhance the external Poisson drive onto these populations parametrized by *κ* (see “Methods”). However, already a perturbation of a few percent leads to a state with unrealistically high rates (Fig 3D), caused by the reduced basin of attraction of the low-activity state similar to Fig 2D. Our aim is to improve the working point of the model such that all populations exhibit spiking activity ≳ 0.05 spikes/s while preventing the model from entering a state with unrealistically high rates of ≳ 30 spikes/s (figure 13 of [25], [26]). The employed technique exposes the mechanism giving rise to the observed instability and identifies the circuitry responsible for this dynamical feature.

### Targeted modifications preserve global stability

We apply the procedure derived in “*Methods*” and find targeted modifications to the connectivity ***K*** that preserve the global stability of the low-activity fixed point for increased values of the external drive *κ*.

**Fig 4:**
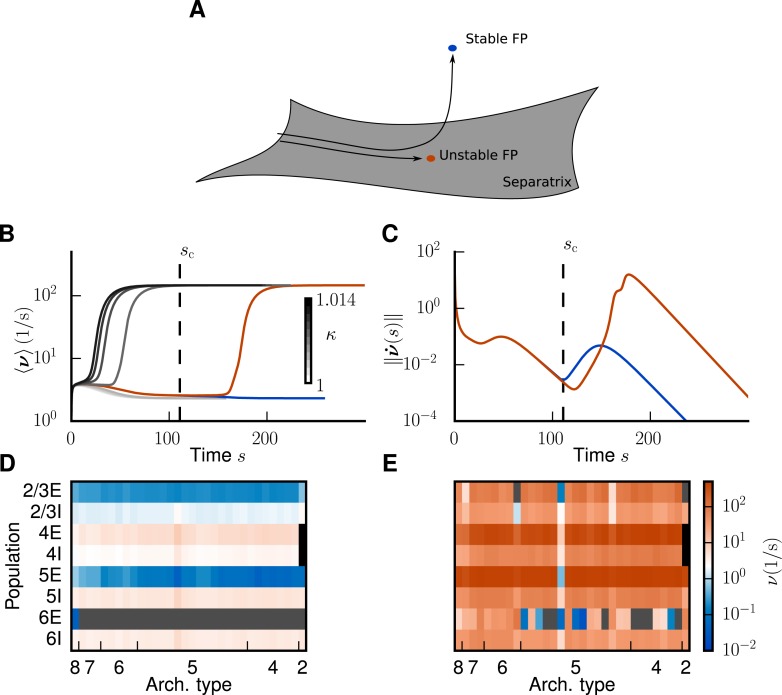
Application of the mean-field theory to the multi-area model. **A** Trajectories of Eq (1) starting inside the separatrix converge to an unstable fixed point. Trajectories starting close to the separatrix are initially attracted by the unstable fixed point but then repelled in the fixed point’s unstable direction and finally converge to a stable fixed point. **B** Firing rate averaged across populations over time. Integration of Eq (1) leads to convergence to either the low-activity (LA) or the high-activity (HA) attractor for different choices of the external input factor κ, with κ=1 the original level of external drive. We show eight curves with κ varying from 1.0 to 1.014 in steps of 0.002 and two additional curves for κ=1.007662217,1.007662218. The curves for the largest factor (κ=1.007662217) that still leads to the LA state and for the smallest factor (κ=1.007662218) that leads to the HA state are marked in blue and red, respectively. The four curves with κ≤1.006 coincide with the blue curve. **C** Euclidean norm of the velocity vector in the integration of Eq (1) for the different choices of κ. The vertical dashed line indicates the time $s_{\text{c}}$ of the last local minimum in the blue curve. **D** Stationary firing rate in the different areas and layers of the model in a low-activity state for κ=1.0 as predicted by the mean-field theory (same display as in Fig 3). **E** As D, but showing the high-activity state for κ=1.125.

In the following we choose the inactive state ***ν***(0) = (0, …, 0)^T^ as the initial condition. The exact choice is not essential since we are only interested in the fixed points of the system. Fig 4B shows the integration of Eq (1) over pseudo-time *s* for different levels of the external drive to populations 5E and 6E parametrized by *κ*. For low values of *κ*, the integration converges to the LA fixed point shown in Fig 4D, and is in agreement with the activity emerging in the simulation (Fig 3C). For increased values of *κ*, the system settles in the HA fixed point (Fig 4E), again in agreement with the simulation. The population-specific firing rates in the HA state found in the mean-field predictions (Fig 4E) are also close to those in the simulation (Fig 3D), but minor deviations occur due to the violation of the assumptions made in the diffusion approximation. In particular, at these pathologically high rates, the neurons fire regularly and close to the reciprocal of their refractory period, while in the mean-field theory we assume Poisson spike statistics. Still, the mean-field theory predicts the bistability found in the simulation. Since the theory yields reliable predictions in both stable fixed points, we assume that also the location of the unstable fixed point in between these two extremes is accurately predicted by the theory.

**Fig 5:**
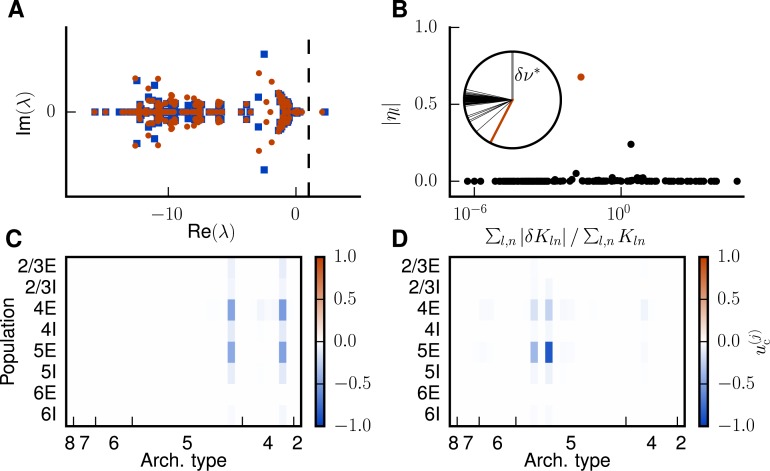
Eigenspectrum analysis of network stability. **A** Eigenvalue spectrum of the effective connectivity matrix M for the first (blue squares) and second (red dots) iteration. The dashed vertical line marks the edge of stability at a real part of 1. **B** Contribution $\eta_{l}$ (Eq (13)) of an individual eigenprojection l to the shift of the unstable fixed point versus the relative change in indegrees associated with l for the first iteration. The data point corresponding to $\lambda_{c}^{\left(1\right)}$ is marked in red. The inset shows the relative angles between ${\delta \nu }^{*}$ and the eigenvectors $u^{l}$. The red line corresponds to the critical eigendirection. **C** Entries of the eigenvector $u_{c}^{\left(1\right)}$ associated with $\lambda_{c}^{\left(1\right)}$ in the populations of the model. The affected areas are 46 and FEF. **D** Same as C for the second iteration.

**Fig 6:**
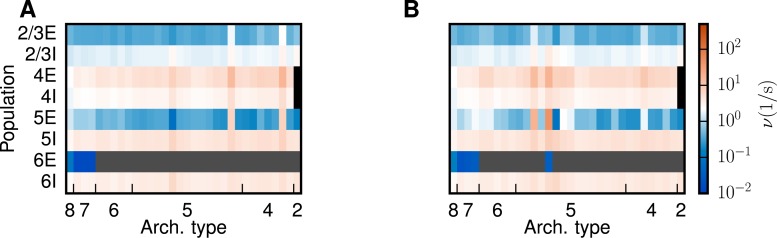
Unstable fixed points in subsequent iterations. Population firing rates at the unstable fixed point as predicted by the mean-field theory encoded in color for iterations 1 (**A**) and 2 (**B**). Same display as in Fig 3.

To control the separatrix we need to find the unstable fixed point of the system. This is nontrivial since the numerical integration of Eq (1) for finding equilibria by construction only converges to stable fixed points. If the unstable fixed point has only one repelling direction (Fig 5A), it constitutes a stable attractor on the *N* − 1 dimensional separatrix. The separatrix is a stable manifold [22], and therefore a trajectory originating in its vicinity but not near an unstable fixed point initially stays in the neighborhood. If an initial condition just outside the separatrix is close to the basin of attraction of a particular unstable fixed point, the trajectory initially approaches the latter. Close to the fixed point the velocity is small. Ultimately trajectories diverge from the separatrix in the fixed point’s unstable direction, as illustrated in Fig 4A. In conclusion, we expect a local minimum in the velocity along the trajectories close to the unstable fixed point. To estimate the location of the unstable fixed point in this manner, we need to find initial conditions close to the separatrix. Naively, we would just fix the value of *κ* and vary the initial condition. However, due to the high dimensionality of our system this is not feasible in practice. Instead, we vary *κ* for a fixed initial condition. Fig 4B shows the firing rate averaged across populations for two trajectories starting close to the separatrix, where the first one converges to the LA fixed point and the second one to the HA state. The trajectories diverge near the unstable fixed point and thus we define the last local minimum of the Euclidean norm of the velocity vector as the critical time *s*_*c*_ at which we assume the system to be close to the unstable fixed point (Fig 4C). We find four relevant and distinct unstable fixed points, of which two are shown in Fig 6.

To counteract the shift of the separatrix caused by the increase in *κ*, we follow the procedure described in “*Methods*”. We subject the modifications of connectivity to the additional following constraints. In line with the anatomical literature, we do not allow for changes of the connectivity that would lead to cortico-cortical connections originating in the granular layer 4 [27], and we also disallow inhibitory cortico-cortical connections, as the vast majority of long-range connections are known to be excitatory [28, 29]. In addition, we naturally restrict indegrees to positive values. We find that four iterations (numbered by index *j*) corresponding to the four distinct unstable fixed points suffice to preserve the basin of attraction of the LA state with respect to an increase of the external drive up to *κ* = 1.15. In the following we concentrate on iterations 1 and 2, where the second one is also representative for iterations 3 and 4, which are qualitatively alike. To derive the required modifications of the indegree matrix, we decompose ***K*** into its *N* eigenmodes and quantify the contribution of each eigenmode to the shift of the unstable fixed point (see “Methods”). This allows us to identify the most effective eigendirection: in each iteration *j* there is exactly one unstable eigendirection with an eigenvalue $Re\left(\lambda_{c}^{\left(j\right)}\right)>1$ (Fig 5A). The associated critical eigenvector is approximately anti-parallel to the shift of the fixed point, δν* (inset of Fig 5B), and of similar length. The critical eigendirection (red dot in Fig 5B) constitutes the most effective modification, giving the largest contribution to the desired shift while requiring only a small change of 2.3% in average total indegrees. In the chosen space of eigenmodes, the modifications are minimal in the sense that only this most effective eigenmode is changed.

**Fig 7:**
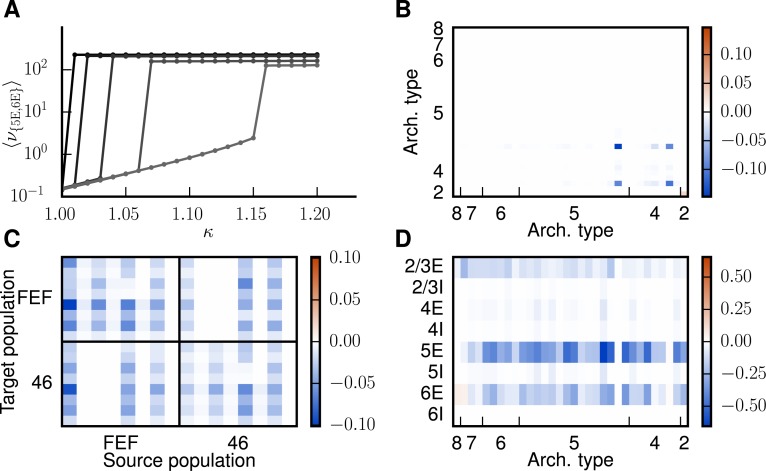
Altered phase space and modified connections. **A** Firing rates averaged across populations 5E and 6E and across areas for different stages from the original model (black) to iteration 4 (light gray) as a function of κ, predicted by the mean-field theory. **B** Relative changes in the indegree $\delta K_{AB}/\sum_{B}\;K_{AB}$ between areas *A*, *B* in the first iteration. **C** Layer-specific relative changes $\delta K_{ln}/\sum_{n}\;K_{ln}$ in the connections within and between areas FEF and 46, for the first iteration. Populations are ordered from 2/3E (left) to 6I (right) on the horizontal axis and from 6I (bottom) to 2/3E (top) on the vertical axis as in panel D. **D** Relative changes in population-specific indegrees summed over target populations, $\sum_{l}\;\delta K_{ln}/\sum_{l}\;K_{ln}$, combined for iterations two, three and four.

The associated eigenvector $u_{c}^{\left(1\right)}$ predominately points into the direction of populations 4E and 5E of areas FEF and 46 (Fig 5C), while $u_{c}^{\left(2\right)}$ has large entries in the 5E populations of two areas (Fig 5D). The high rates of these populations at the unstable fixed points (cf. Figs 6A and 6B with 5C and 5D) reflect that the instability is caused by increased rates in excitatory populations, particularly in population 5E. Each iteration shifts the transition to the HA state (the value of *κ* for which the separatrix crosses the initial condition) to higher values of *κ* and increases the attainable rates of populations 5E and 6E in the LA state (Fig 7A). After all four iterations, the average total indegrees (summed over source populations) of the system are changed by 11.3%. The first iteration mainly affects connections within and between areas 46 and FEF (Fig 7B). In particular, the excitatory loops between the two areas are reduced in strength, especially those involving layer 5 (Fig 7C). We thus identify two areas forming a critical loop. In the remaining iterations, the changes are spread across areas and especially connections originating in layer 5 are weakened (Fig 7D). In conclusion, the method identifies critical structures in the model both on the level of areas and on the level of layers and populations, and leads to a small but specific structural change of the model.

### Analysis of the modifications

**Fig 8:**
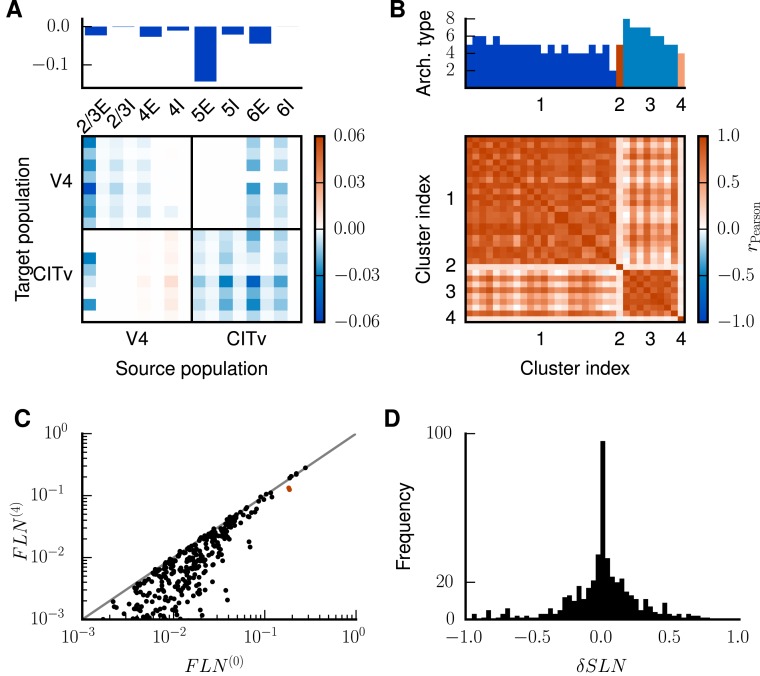
Analysis of changes in connectivity. **A** Top panel: relative changes in population-specific intrinsic indegrees summed over target populations and averaged across areas, $\langle \sum_{i}\delta K_{AA}^{ij}/\sum_{i}K_{AA}^{ij}\rangle_{A}$. Bottom panel: changes in the indegrees within and between exemplary areas V4 and CITv relative to the total indegrees of the target populations, i.e., $\delta K_{AA}^{ij}/\sum_{j}K_{AA}^{ij}$. Populations ordered as in Fig 7C. **B** Pearson correlation coefficient of the changes of the internal indegrees $\delta K_{AA}^{ij}$ between all pairs of the 32 areas. Areas ordered according to hierarchical clustering using a farthest point algorithm [31]. The heights of the bars on top of the matrix indicate the architectural types of the areas (types 1 and 3 do not appear in the model) with color representing the respective clusters. **C** FLN of the modified connectivity after 4 iterations versus the original FLN of the model. Only $FLN>10^{-3}$ are shown for a better overview. The overlapping red dots represent the connections between areas 46 and FEF. Unity line shown in gray. **D** Histogram of the cumulative changes in SLN over all four iterations $\left(\delta SLN=SLN^{\;(4)}-SLN^{\;(0)}\right)$.

In the following we analyze the modifications of the connectivity with respect to the internal and inter-area connections in detail. The intrinsic circuits of the areas are modified in different directions, as shown for two exemplary areas V4 and CITv in Fig 8A. Despite this heterogeneity, significant changes affect mostly excitatory-excitatory connections (Fig 8A, bottom panel) with connections from population 5E experiencing the most significant changes (top panel of Fig 8A). In fact, the anatomical data [30] underlying the microcircuit model [23] contain only two reconstructed excitatory cells from layer 5, but considerably more for other cell types, indicating a higher uncertainty for layer 5 connections. Fig 8B shows the correlation between intrinsic connectivity changes for all pairs of areas, with areas ordered according to a hierarchical clustering using a farthest point algorithm [31] on the correlation matrix. We find four clusters each indicating a group of areas which undergo changes with similar patterns. The groups are displayed in different colors in the histogram in Fig 8B. The areas of the model are categorized into architectural types based on cell densities and laminar thicknesses (see “Methods”). Areas with architectural type 4, 5 and 6 are distributed over several clusters. We can interpret this as a differentiation of these types into further subtypes. The resulting changes of the intra-areal connectivity are small (Fig 8A), but still significant for network stability.

Connections between areas can be characterized by their *FLN* and *SLN* (see “Methods”). The *FLN* reflects the overall strength of an inter-areal connection and is only weakly affected across connections (Fig 8C), with a correlation between original and modified logarithms of *FLN* of *r*_Pearson_ = 0.79. Significant variations in the *FLN* occur mostly for very weak connections that are likely to have substantial relative uncertainties in the experimental data. The two overlapping red dots in Fig 8C represent the connections between areas 46 and FEF, which are modified in the first iteration (Fig 7C). The *SLN* determines the laminar pattern of the location of source neurons for cortico-cortical connections. Overall, data are available for 24% of the inter-areal connections in the parcellation of Felleman & van Essen [27], while the *SLN* for the rest are derived from the sigmoidal law. The majority of connections undergo small changes in their laminar source pattern (Fig 8D) and connections with large modifications (|*δSLN*| > 0.5) are weak (average $\bar{FLN}=6\cdot 10^{-4}$ compared to $\bar{FLN}=10^{-2}$ in the model as a whole). Because weak connections are represented by low counts of labeled neurons, they have a relatively large uncertainty in their laminar patterns, justifying larger adjustments. Spearman’s rank correlation between the *SLN* of the original model that were directly taken from experiments and the logarithmic ratios of cell densities is *ρ* = −0.63 (*p* = 3 ⋅ 10^−11^, p-value of a two-sided test for uncorrelated data). For the modified model, we take the *SLN* of all connections into account and obtain *ρ* = −0.40 (*p* = 6 ⋅ 10^−20^), indicating a reduced, but still significant, monotonic dependence between *SLN* and the logarithmic ratios of cell densities.

To judge the size of the modification to the connectivity, we compare it to the variability of measured cortico-cortical connection densities [1]. We quantify the latter as the average inter-individual standard deviation of the logarithmic *FLN*, i.e., $\sigma =\langle \sqrt{\bar{{\left(\text{log}FLN-\bar{\text{log}FLN}\right)}^{2}}}\rangle$, where the overbar $\bar{\cdot }$ denotes the average over injections and 〈⋅〉 the average over connections. This variability equals 2.17 while the average modification of the logarithmic *FLN* is 1.34. The main experimental connection probabilities used to construct the intra-areal connectivity of the model have an average relative standard deviation of 30% across electrophysiological experiments (cf. Table 1 of [23]) while the intra-areal connection probabilities of the model are modified by 9% on average. The authors of [32] report even greater variability in their review on cortico-cortical and thalamocortical connectivity. These considerations show that on average, the changes applied to the connectivity are well within the uncertainties of the data. Overall, 35 out of 603 connections were removed from the network. In the CoCoMac database, 83% of these are indicated by only a single tracer injection, while the overall proportion of connections measured by a single injection is 59%.

**Fig 9:**
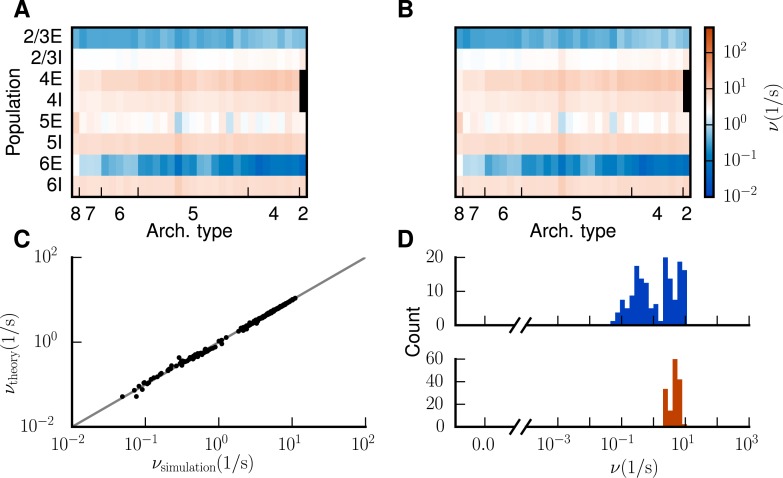
Improved low-activity fixed point of the model. Population-averaged firing rates for κ=1.125 encoded in color (**A**) predicted by the analytical theory and (**B**) obtained from the full simulation of the spiking network. Same display as in Fig 3. **C** Analytical versus simulated firing rates (black dots) and identity line (gray). **D** Histogram of population-averaged simulated firing rates. Same display as in Fig 3.

For the modified connectivity and *κ* = 1.125, which we choose to avoid being too close to the transition (Fig 7A), the theory predicts average rates in populations 5E and 6E of 1.3 and 0.18 spikes/s, which is closer to experimentally observed rates compared to the original model. Furthermore we find that the modified connectivity allows us to decrease the inhibition in the network to *g* = −11. Simulating the full spiking network model then results in reasonable rates across populations and areas (Fig 9B and 9D). The average rates in populations 5E and 6E are increased compared to a simulation of the original model from 0.09 and 2 ⋅ 10^−5^ spikes/s to 3.0 and 0.4 spikes/s, respectively. All populations exhibit firing rates within a reasonable range of 0.05 to 30 spikes/s (Fig 9D), as opposed to the original state in which a considerable fraction of excitatory neurons is silent (Fig 3E). The theoretical prediction is in excellent agreement with the rates obtained in the simulation (Fig 9A and 9C). Small discrepancies are caused by violations of the employed assumptions, i.e., Poissonian spiking statistics [33]. Differences between theory and simulation are small, and negligible for the central aim of the study: the integration of activity constraints into the data-driven construction of multi-scale neuronal networks.

## Discussion

This study investigates the link between experimentally measured structural connectivity and neuronal activity in a multi-scale spiking network model of the vision-related areas of macaque cortex [24]. We devise a theoretical method that systematically combines anatomical connectivity with physiological activity constraints. Already weak constraints, demanding the activity to neither vanish nor be pathologically high, yield a set of specific but small structural modifications necessary to increase the model’s excitation to a realistic level. We do not fit the model parameters to experimental activity data in the sense of minimizing an error function, since the sparse relevant experimental data do not allow for defining such a function in a meaningful way. Nevertheless, we considerably improve the network activity with a change in only a small set of parameters. The procedure constrains the experimentally obtained connectivity maps to a realization that is compatible with physiological experiments. This establishes a path from experimentally observed activity to specific hypotheses about the anatomy and demands for further experiments.

Connections are modified both inside and between cortical areas, on average well within the known uncertainties of the underlying data. The model areas are based on the early sensory cortex model presented in [23]. This circuit is adapted to individual areas by taking into account neuronal densities and laminar thicknesses. The model definition renders areas with equal architectural type similar in their internal connectivity, a drastic but inevitable simplification due to the lack of more detailed experimental data. The proposed method softens this assumption: small adaptations of internal connectivity distinguish the architectural types into further subtypes. These modifications are significant for the global stability of the network. Thus, our approach enables purely anatomy-based area categorizations to be refined with dynamical information.

Connections between areas are changed in terms of total strength and laminar patterns. Overall, the changes are small, but significant for specific connections. The loop formed by areas 46 and FEF is critical to the global stability of the network. Both areas have been investigated in [1], albeit in a different parcellation scheme than the scheme used here [27]. Our method suggests a weaker coupling of these two areas than found in the anatomical data set. Uncertainties, partly due to the mapping between parcellations, leave room for this interpretation. Areas 46 and FEF belong to prefrontal cortex and are multimodal, indicating that the influence of other parts of cortex could stabilize them, a mechanism outside the scope of the present model of vision-related areas. Both explanations can be tested either in an experimental study or with an extended model.

A few weak connections undergo large changes in their laminar patterns. With the present activity constraints, the method hereby weakens hierarchical relations in the model structure, indicating that preserving these relations requires additional dynamical constraints such as layer-specific coherence between areas [34, 35]. Conversely, it is possible to achieve satisfactory population rates with a less pronounced hierarchical structure.

Our analysis reveals that layer 5 excitatory cells play a critical role in the model’s dynamics, in line with the observed ongoing activity in mammalian neocortex [36, 37]. This critical role is often attributed to single-neuron properties, with a subset of layer 5 neurons displaying pacemaker activity [38–40]. In addition, we here find that the network architecture itself already explains the strong impact of layer 5 on the phase space of the network, suggesting that single-neuron properties and network structure jointly enable layer 5 to exert its dominant influence.

The cortico-cortical connectivity of the model is compiled from the extensive dataset of [1] combined with the CoCoMac database [41, 42], which collects data from hundreds of tracing studies. One could consider alternative methods for combining this information into one connectivity graph, for instance taking into account how consistently a given connection is reported across studies [43], and compare different methods by analyzing the resulting network dynamics. The presented mean-field theory could then be used to estimate the firing rates of each network instance without performing time-consuming simulations. However, here we first choose the integrative approach that accumulates all available experimental evidence into a single model and afterwards modify the resulting connectivity with our analytical procedure, thereby effectively discarding uncertain connections.

We restrict this study to networks of leaky integrate-and-fire model neurons, consistent with the key concept of the models we consider: individual cells are modeled in a simple manner to expose the impact of structural connectivity on the network dynamics. Moreover, the current-based leaky integrate-and-fire neuron can reproduce in-vivo like activity [44, 45] and is analytically tractable, which enables the identification of mechanisms underlying specific network effects. More complex neuron models can be incorporated into the method by replacing the gain function of each neuronal population with an analytical expression or an interpolated function obtained from spiking single-neuron simulations. For example, one could use a conductance-based point-neuron model for which the network dynamics can be described by population rate models [46], featuring a non-monotonic gain function: the gain is reduced if excitatory and inhibitory inputs are increased in a balanced manner [47]. Generally, this renders a system more stable. However, the bistability considered in our work is caused by excitatory inputs. Since conductance-based models also have a monotonically increasing gain function in dependence of the excitatory conductance alone, we expect the bistability to occur for such models as well.

Importantly, our method only requires a description of the system’s fixed points and their dependence on model parameters. The employed theory uses the diffusion approximation to derive a self-consistency equation for the stationary population rates valid for high indegrees and small synaptic efficacies. These requirements are fulfilled in the multi-area model and therefore the theoretical prediction agrees with the stationary activity of the simulation. Moreover, the theory predicts the bistability of the model, which is non-trivial, as the mean-field assumption of Poisson statistics of the activity is generally violated in the high-activity state. Nevertheless, since the activity in this state is mostly driven by strong mean inputs and the theory converges to the noiseless solution in the mean-driven limit, its predictions still provide viable approximations. The firing rates in the unstable fixed points are predominantly low, while the exceptions with very high rates are again mean-dominated. Presumably this explains the accuracy by which the theory predicts the locations of these fixed points and the resulting global stability properties.

Since the high-activity attractor of the model under consideration is unrealistic, we aim to prevent a transition to this state. However, in the high-dimensional system it is not a priori clear in which direction the separatrix has to be shifted to ensure stable dynamics. We therefore choose a pragmatic approach and shift the separatrix back to its initial location, inverting the shift which reduced the global stability of the low-activity fixed point. We achieve this to a good approximation by preserving the location of unstable fixed points on the separatrix. To this end, we use a linearization around these locations and an eigenmode decomposition to identify the set of connections to adjust. In the multi-area model, this linearization is justified because the system operates close to an instability so that only minor modifications are required. The method can be generalized to larger modifications by changing parameters iteratively in small steps.

Biological networks have various stabilization mechanisms not considered here, which render them less critical. For instance, during growth, homeostatic mechanisms guide the system toward the right structure. Furthermore, short-term synaptic plasticity (reviewed in [48]), homeostatic synaptic scaling [49] and spike-frequency adaptation (e.g. summarized in [50]) may prevent the system from entering the high-activity state. However, introducing these self-organizing mechanisms increases model complexity, causing a more intricate relation between structure and activity. Therefore, we start from a mean-field description on the level of neuronal populations, ignoring details of synaptic dynamics. Mild constraints on the activity lead to a network structure within the anatomical range of parameters. This network yields globally stable activity, suggesting that additional stabilization mechanisms are not required to achieve this. Nonetheless, they can potentially render the network more robust against external stimulation.

The observed inter-individual variability may reflect that mechanisms of self-organization and homeostasis find structurally different implementations of the same function. Thus, in studies across individuals we cannot expect that progress in experimental technology narrows down the variability of parameters indefinitely. The combined ranges of parameters rather specify the solution space, and our method provides a way to find a particular solution.

In principle, the method applies to any model parameter. It would be possible to modify, for example, synaptic weights. Since experimental data on synaptic weights are sparse, this is another natural choice. Moreover, such an analysis may provide hints about suitable synaptic plasticity rules that dynamically stabilize the model. The method can be applied to networks with more complex sets of attractors compared to the bistable case considered here. Though in high-dimensional systems such as our multi-area model, a larger number of attractors would make it more challenging to find all relevant unstable fixed points, the underlying idea of preserving the location of a separatrix is general. In contrast to the model considered here, transitions between fixed points can have a functional meaning in certain neuronal networks with multiple attractors. The specific location of the separatrix is then functionally relevant. Our method exposes the sensitivity of the location of the separatrix to certain model parameters and allows controlling its location in a specific manner.

In this work, we analyzed the global stability properties of the neuronal network on the population level. In contrast, Ostojic [51] performs a local stability analysis on the level of single neurons of an initially stable fixed point in a system with only one attractor. The author investigates the point at which the real parts of the eigenvalues of the Jacobian matrix evaluated at this fixed point become positive, i.e., the fixed point turns spectrally unstable and the system undergoes a transition to a heterogeneous asynchronous state. Analyzing the spectral stablilty on the single-neuron level does not reveal the global stability properties required in the current work: While a local stability analysis only considers infinitesimal perturbations, studying the basin of attraction gives information about the size of fluctuations against which the fixed point is stable. We expect both attractors to be spectrally stable because they do not show strong rate fluctuations and the mean-field theory predicts the activity accurately in both cases. Nevertheless, a heterogeneous state could occur if the synaptic weights were increased or the external drive was stronger. However, [52] show that the transition in stochastic systems that quantitatively resemble the spiking network [53], does not coincide with the loss of spectral stability.

One striking feature of the heterogeneous state is bursty spiking behavior of individual cells. Bursty spiking is also observed in the multi-area model for increased synaptic weights of inter-area connections [24]. The fixed points cannot be accurately described in this case because the fluctuations need to be taken into account [54]. Simulations show (figures 4 and 5 of [24]) that the modifications obtained in this study are still able to prevent the system from a transition to a HA attractor also in the presence of bursting neurons. This indicates that the phase space does not change qualitatively and our results are robust against such bursting behavior.

Experimental data on stationary activity in cortex are sparse. We therefore restrict ourselves to fundamental constraints and increase the drive to the model in an area-unspecific way to fulfill them. The resulting heterogeneity of the firing rates across areas is thus not imposed by the method, but rather arises from the connectivity that remains strongly informed by anatomical data. Alternatively, one could predefine a desired state and investigate the parameter changes necessary to achieve it.

The presented analytical method that combines anatomy and activity data into a consistent model is restricted to stationary firing rates. In future studies, also higher-order statistical measures of activity can be used as constraints. Resting-state fMRI, for example, provides information on the functional connectivity between areas as a second-order measure. When combined with analytical predictions of functional connectivity, the method may shed light on the anatomical connection patterns underlying inter-area communication.

## Methods

In this study, we model single cells as leaky integrate-and-fire model neurons with exponentially decaying postsynaptic currents. Table 1 specifies the model parameters.

**Table 1:** Specification of the neuron and synapse parameters.

**Synapse parameters**		
**Name**	**Value**	**Description**
J±δJ	87.8 ± 8.8 pA	excitatory synaptic strength
g	−16 (Fig 3) −11 (Fig 9)	relative inhibitory synaptic strength
$d_{e}\pm \delta d_{e}$	1.5 ± 0.75 ms	local excitatory transmission delay
$d_{i}\pm \delta d_{i}$	0.75 ± 0.375 ms	local inhibitory transmission delay
d±δd	$d=s/v_{t}\pm \frac{1}{2}s/v_{t}$	inter-areal transmission delay, with *s* the distance between areas
$v_{t}$	3.5 m/s	transmission speed
**Neuron model**		
**Name**	**Value**	**Description**
$\tau_{m}$	10 ms	membrane time constant
$\tau_{r}$	2 ms	absolute refractory period
$\tau_{s}$	0.5 ms	postsynaptic current time constant
$C_{m}$	250 pF	membrane capacity
$V_{r}$	−65 mV	reset potential
θ	−50 mV	fixed firing threshold
$E_{L}$	−65 mV	leak potential

In the diffusion approximation, the dynamics of the membrane potential *V* and synaptic current *I*_s_ is [33]

$$\begin{array}{lll} \tau_{\text{m}}\frac{\text{d}V}{\text{d}t} & = & -V+I_{\text{s}}(t)\\ \tau_{\text{s}}\frac{\text{d}I_{\text{s}}}{\text{d}t} & = & -I_{\text{s}}+\mu +\sigma \sqrt{\tau_{\text{m}}}\xi (t), \end{array}$$ (2)

where *τ*_m_ is the membrane time constant and *τ*_s_ the synaptic time constant, respectively. The membrane resistance *τ*_m_/*C*_m_ has been absorbed into the definition of the current. The input spike trains are approximated by a white noise current with fluctuations ∝ *σ*^2^ and mean value *μ*. Here *ξ* is a centered Gaussian white process satisfying 〈*ξ*(*t*)〉 = 0 and 〈*ξ*(*t*)*ξ*(*t*′)〉 = *δ*(*t* − *t*′). Whenever the membrane potential *V* crosses the threshold *θ*, the neuron emits a spike and *V* is reset to the potential *V*_r_, where it is clamped during *τ*_r_. All neurons in one population have identical parameters, so that we can describe the network activity in terms of population-averaged firing rates *ν*_*i*_ that depend on population-dependent input *μ*_*i*_, *σ*_*i*_ determined by the connectivity. Using the Fokker-Planck formalism, the stationary firing rates for each population *i* are given by [33]

$$\begin{array}{lll} \frac{1}{\nu_{i}} & = & \tau_{\text{r}}+\tau_{\text{m}}\sqrt{\pi }\int_{\frac{V_{r}-\mu_{i}(A)}{\sigma_{i}(A)}+\gamma \sqrt{\frac{\tau_{\text{s}}}{\tau_{\text{m}}}}}^{\frac{\theta -\mu_{i}(A)}{\sigma_{i}(A)}+\gamma \sqrt{\frac{\tau_{\text{s}}}{\tau_{\text{m}}}}}{\text{e}}^{x^{2}}\;(1+\text{erf}(x))\text{d}x\\ & ≕ & 1/\Phi_{i}(\nu ,A) \end{array}$$ (3)

$$\mu_{i}\left(A\right)=\tau_{m}\sum_{j}K_{ij}J{}_{ij}\nu_{j}+\tau_{m}K_{\text{ext}}J_{\text{ext}}\nu_{\text{ext}}$$ (4)

$$\sigma_{i}^{2}\left(A\right)=\tau_{m}\sum_{j}K_{ij}J_{ij}^{2}\nu_{j}+\tau_{m}K_{\text{ext}}J_{\text{ext}}^{2}\nu_{\text{ext}},$$ (5)

which is correct up to linear order in $\sqrt{\tau_{s}/\tau_{m}}$ and where $\gamma =|\zeta (1/2)|/\sqrt{2},$ with *ζ* denoting the Riemann zeta function [55]. Here, ***A*** is chosen from the set of model parameters {***K***, ***J***, *ν*_ext_, …}. If ***A*** is a matrix, we vectorize it by concatenating its rows and indicate this by lower case, i.e., ***a*** = (*a*_00_, *a*_01_, …, *a*_0*N*_, *a*_10_, …, *a*_1*N*_, *a*_*N*0_ …, *a*_*NN*_) = vec(***A***^T^)^T^ following [56]. If the chosen parameter is a scalar we denote it with *a*.

We find the fixed points of Eq (3) by solving the first-order differential equation Eq (1) [57]

$$\dot{\nu }≔\frac{d\nu }{ds}=\Phi \left(\nu ,A\right)-\nu ,$$

using the classical fourth-order Runge-Kutta method (RK4) with step size *h* = 0.01, where *s* denotes a dimensionless pseudo-time. The same approach can be used to solve the activity on a single neuron level [58]. Note that Eq (1) does not reflect the real time evolution of the population rates, but rather is a mathematical method to obtain the system’s fixed points. In contrast to [57] we do not only search for stable fixed points, but also use Eq (1) to obtain unstable attractors (cf. “Results”), an idea originating from the study of simple attractor networks ([59] esp. their figure 2 and eq 12).

In a bistable situation, the initial condition of Eq (1) determines which fixed point the system settles in. However, studying the behavior for a particular initial condition is of minor interest, since the actual spiking network is a stochastic system which fluctuates around the fixed points of the deterministic system defined by Eq (1). Even if we knew that Eq (1) would relax to the LA fixed point for one particular initial condition, this would not necessarily imply that this state is indefinitely stable. Global stability is determined by the size of the basin of attraction of the LA fixed point.

In the following, we derive the equations leading us to targeted modifications of a parameter ***b*** necessary to compensate for the changes in the global stability induced by the change of another parameter ***a***. To this end, we study the behavior of the fixed points with respect to an infinitesimal change ***δa* = *a*′ − *a*** in the chosen model parameter. Let ν*(***a***) and ν*(***a*′**) be the corresponding locations of the fixed points and δν* = ν*(***a*′**) − ν*(***a***) their separation. We can then expand ν*(***a*′**) into a Taylor series up to first order in ***δa*** and obtain

$$\begin{array}{lll} \nu^{*}(a^{\prime }) & = & \nu^{*}(a)+\Delta_{a}\delta a\\ \Leftrightarrow \delta \nu^{*} & = & \Delta_{a}\delta a, \end{array}$$ (6)

with

$$\begin{array}{lll} \Delta_{a,ij} & = & \frac{\text{d}\nu_{i}\left(\mu_{i},\sigma_{i}\right)}{\text{d}a_{j}}\\ & = & \frac{\text{d}\Phi_{i}\left(\mu_{i},\sigma_{i}\right)}{\text{d}a_{j}}\\ & = & \underset{S_{i}}{\underset{︸}{\frac{\partial \Phi_{i}}{\partial \mu_{i}}}}\frac{\text{d}\mu_{i}}{\text{d}a_{j}}+\underset{T_{i}}{\underset{︸}{\frac{\partial \Phi_{i}}{\partial \sigma_{i}}\frac{1}{2\sigma_{i}}}}\frac{\text{d}\sigma_{i}^{2}}{\text{d}a_{j}}, \end{array}$$ (7)

where we notice that *S*_*i*_ and *T*_*i*_ only depend on the target population *i*. We accordingly define two diagonal matrices ***S*** and ***T*** with *S*_*ii*_ = *S*_*i*_ and *T*_*ii*_ = *T*_*i*_. We further define the connectivity matrix **W** = ***K*** ⊛ **J**, where ⊛ denotes element-wise multiplication, also called the Hadamard product (see [60], for a consistent set of symbols for operations on matrices). The derivatives with respect to *a*_*j*_ have the compact expressions

$$\begin{array}{lll} \frac{\text{d}\mu_{i}}{\text{d}a_{j}} & = & \frac{\partial \mu_{i}}{\partial a_{j}}+\sum_{n}\frac{\partial \mu_{i}}{\partial \nu_{n}}\frac{\text{d}\nu_{n}}{\text{d}a_{j}}\\ & = & {\left(D_{a}\mu \right)}_{ij}+\tau_{\text{m}}\sum_{n}(\underset{=W_{in}}{\underset{︸}{K_{in}J_{in}}})\Delta_{a,nj}, \end{array}$$

with the Jacobian ${\left({\text{D}}_{a}f\right)}_{ij}≔\frac{\partial f_{i}}{\partial a_{j}}$ of some vector-valued function **f** and

$$\frac{d\sigma_{i}^{2}}{da_{j}}={\left(D_{a}\sigma^{2}\right)}_{ij}+\tau_{m}\sum_{n}\left(\underset{=W_{2,in}}{\underset{︸}{K_{in}J_{in}^{2}}}\right)\Delta_{a,nj}\;,$$

where we use the Hadamard product again to define the matrix **W_2_** ≔ **K** ⊛ **J** ⊛ **J**. Inserting the total derivatives into Eq (7), we derive the final expression for **Δ_*a*_**, reading

$$\begin{array}{llll} & \Delta_{a} & = & S[D_{a}\mu +\tau_{\text{m}}W\Delta_{a}]+T[D_{a}\sigma^{2}+\tau_{\text{m}}W_{2}\Delta_{a}]\\ \Leftrightarrow & \Delta_{a} & = & {\left[1-\underset{≕M}{\underset{︸}{\tau_{\text{m}}(SW+TW_{2})}}\right]}^{-1}\underset{≕{\bar{\Delta }}_{a}}{\underset{︸}{\left[SD_{a}\mu +TD_{a}\sigma^{2}\right]}}, \end{array}$$ (8)

where we use 1 for the identity matrix and define the effective connectivity matrix ***M*** and the matrix ${\bar{\Delta }}_{a}$. The latter has dimensionality *N* × *P*, where *P* is the dimension of ***a*** (for example, *P* = *N*^2^ for ***a*** = ***k*** the vector of indegrees). With the aid of Eq (8), evaluating Eq (6) at the unstable fixed point predicts the shift of the separatrix (Fig 2D) to linear order. We now consider an additional parameter ***b*** which is modified to counteract the shift of the unstable fixed point caused by the change in parameter ***a***, i.e.,

$$\begin{array}{llll} & \Delta_{a}\delta a & \overset{!}{=} & -\Delta_{b}\delta b\\ \Leftrightarrow & {\bar{\Delta }}_{a}\delta a & = & -{\bar{\Delta }}_{b}\delta b, \end{array}$$ (9)

where we used that the inverse of 1 − M appears on both sides of the equation and hence drops out. Note that the tuple (***a***, ***b***) may represent any combination of model parameters, for example external input, indegrees, synaptic weights, etc. For a particular choice of (***a***, ***b***) we solve Eq (9) for ***δb***. For the illustrative example shown in Fig 2, where *a* = *ν*_ext_ and *b* = *K*, Eq (9) simplifies to

$$\delta K=\frac{{\bar{\Delta }}_{\nu_{\text{ext}}}\delta \nu_{\text{ext}}}{{\bar{\Delta }}_{K}}=\frac{K_{\text{ext}}\delta \nu_{\text{ext}}}{\nu },$$

since *S* and *T* appearing in the respective ${\bar{\Delta }}^{\prime }$s cancel each other.

To determine critical connections in a more complex model, we choose ***b*** = ***k***, i.e. the vector of indegrees, and solve Eq (9) with a decomposition into eigenmodes. We can write the right-hand side as

$$-{\left({\bar{\Delta }}_{k}\delta k\right)}_{i}=-\sum_{j}\tau_{m}\left(S_{i}J_{ij}+T_{i}J_{ij}^{2}\right)\nu_{j}\delta K_{ij}\;.$$ (10)

The equation holds because *μ*_*i*_, *σ*_*i*_ are only affected by connections to population *i*, and therefore their derivatives ∂*μ*_*i*_/∂*k*_*l*_, ∂*σ*_*i*_/∂*k*_*l*_ and hence ${\bar{\Delta }}_{k,il}$, vanish for *l* ∉ [(*i* − 1)*N* + 1, *iN*]. We now make the ansatz

$$\delta K_{ij}=\sum_{l}\frac{\epsilon_{l}}{\tau_{\text{m}}}\frac{{\left(u^{l}v{}^{lT}\right)}_{ij}}{\left(SJ+T(J⊛J)\right){}_{ij}}\;,$$ (11)

which decomposes the changes ***δK*** into eigenmodes of the effective connectivity. The ***u***^*l*^ and ***v***^*l*^ are the *l*-th right and left eigenvectors of ***M*** as defined in Eq (8), fulfilling the bi-orthogonality ***v***^*l*T^ ***u***^*n*^ = *δ*_*ln*_ and the completeness relation $\sum_{l}u^{l}v^{l\text{T}}=1$. Inserting Eq (10) with Eq (11) into Eq (9) yields

$${\bar{\Delta }}_{a}\delta a=-\sum_{l}\epsilon_{l}u^{l}v^{l\text{T}}\nu .$$ (12)

Thus we can solve for *ϵ*_*l*_ by multiplying from the left with ***v***^*n*T^

$$\begin{array}{lll} \underset{≕{\hat{a}}^{n}}{\underset{︸}{v^{n\text{T}}{\bar{\Delta }}_{a}\delta a}} & = & -\underset{l}{\sum }\epsilon_{l}\underset{\delta_{nl}}{\underset{︸}{v^{n\text{T}}u^{l}}}\underset{≕{\hat{\nu }}^{l}}{\underset{︸}{v^{l\text{T}}\nu }}\\ \epsilon_{n} & = & -\frac{{\hat{a}}^{n}}{{\hat{\nu }}^{n}}. \end{array}$$

Our goal is to find a set of connections which dominate the size of the basin of attraction of the LA fixed point. Inserting Eq (12) into Eq (6) leads to

$$\begin{array}{lll} \delta \nu^{*} & = & \sum_{l}{\left(1-M\right)}^{-1}\epsilon_{l}u^{l}v^{l\text{T}}\nu \\ & = & \sum_{l}\frac{\epsilon_{l}}{1-\lambda_{l}}u^{l}{\hat{\nu }}^{l}\\ & = & \sum_{l}\frac{-{\hat{a}}^{l}}{1-\lambda_{l}}u^{l}, \end{array}$$

where *λ*_*l*_ are the eigenvalues of ***M***, which are either real or complex conjugate pairs since $M\in {\mathbb{R}}^{N\times N}$. To determine the influence of each eigenmode on the shift of the fixed point, we project the eigenvectors ***u***^*l*^ onto the fixed-point shift δν* by multiplying each side with δν*δν*^T^ and solve again for δν* to obtain

$$\delta \nu^{*}=\sum_{l}\underset{≕{\tilde{\eta }}_{l}}{\underset{︸}{\frac{-{\hat{a}}^{l}}{1-\lambda_{l}}\frac{\delta \nu^{*}{}^{\text{T}}u^{l}}{\delta \nu^{*}{}^{\text{T}}\delta \nu^{*}}}}\delta \nu^{*},$$ (13)

where we define the (possibly complex-valued) coefficients ${\tilde{\eta }}_{l}$. We aim at a decomposition of δν* into real components. If Im(*λ*_*l*_) = 0, ${\tilde{\eta }}_{l}$ is real, so we can work directly with $\eta_{l}≔{\tilde{\eta }}_{l}$. Complex eigenvalues Im(*λ*_*l*_) ≠ 0 and corresponding eigenvectors come in conjugate pairs, so in this case we combine the corresponding coefficients $\eta_{l}≔{\tilde{\eta }}_{l}+{\tilde{\eta }}_{l}^{*}$, to have all contributions $\eta_{l}\in \mathbb{R}$ and ∑_*l*_ *η*_*l*_ = 1 by construction Eq (13). Each *η*_*l*_ quantifies how much of the total fixed-point shift can be attributed to the *l*-th eigenmode, which allows identification of the most effective eigendirection (see “Results”), where we apply the ansatz Eq (11) to a multi-area, multi-layer model of the vision-related areas of macaque cortex.

The spiking simulations of the network model were carried out on the JUQUEEN supercomputer [61]. All simulations were performed with NEST version 2.8.0 [62] with optimizations for the use on the supercomputer which will be included in a future release. The simulations use a time step of 0.1 ms and exact integration for the subthreshold dynamics of the leaky integrate-and-fire neuron model (reviewed in [63]).

### Multi-area model

The multi-area model of the vision-related areas of macaque cortex uses the microcircuit model of [23] as a prototype for all 32 areas in the FV91 parcellation [27] and customizes it based on experimental findings on cortical structure. From anatomical studies, it is known that cortical areas in the macaque monkey are heterogeneous in their laminar structure and can be roughly categorized into 8 different architectural types based on cell densities and laminar thicknesses. This distinction was originally developed for prefrontal areas [64], and then extended to the entire cortex [65]. The visual cortex, and thus the model, comprises areas of categories 2, 4, 5, 6, 7 and 8. Precise layer-specific neuron densities are available for a number of areas, while for other areas, the neuron density is estimated based on their architectural type (see [24] for details).

The inter-areal connectivity is based on binary data from the CoCoMac database [27, 41, 42, 66, 67] indicating the existence of connections, and quantitative data from [1]. The latter are retrograde tracing data where connection strengths are quantified by the fraction of labeled neurons (*FLN*) in each source area. The original analysis of the experimental data was performed in the M132 atlas [1]. Both the FV91 and the M132 parcellations have been registered to F99 space [68], a standard macaque cortical surface included with the software tool Caret [69]. This enables mapping between the two parcellations.

On the target side, we use the exact coordinates of the injections to identify the equivalent area in the FV91 parcellation. To map the data on the source side from the M132 atlas to the FV91 parcellation, we count the number of overlapping triangles on the F99 surface between any given pair of regions and distribute data proportionally to the amount of overlap using the F99 region overlap tool on http://cocomac.g-node.org. In the model, this *FLN* is mapped to the indegree *K*_*AB*_ the target area *A* receives from source area *B* divided by its total indegree, i.e., $FLN_{AB}=K_{AB}/\sum_{B^{\prime }}\;K_{AB^{\prime }}$. Here, *K*_*AB*_ is defined as the total number of synapses between *A* and *B* divided by the total number of neurons in *A*. On the source side, laminar connection patterns are based on CoCoMac [27, 67, 70–73] and on fractions of labeled neurons in the supragranular layers (*SLN*) [2]. Gaps in these data are bridged exploiting a sigmoidal relation between *SLN* and the logarithmized ratio of overall cell densities of the two areas, similar to [74]. We map the *SLN* to the ratio between the number of synapses originating in layer 2/3 and the total number of synapses between the two areas, assuming that only excitatory populations send inter-area connections, i.e., $SLN_{AB}=\sum_{i}K_{AB}^{i,2/3E}N_{A}^{i}/\sum_{i,j}K_{AB}^{ij}N_{A}^{i}$, where the indices *i* and *j* go over the different populations within area *A* and *B*, respectively. In the context of the model, we use the terms *FLN* and *SLN* to refer to the relevant relative indegrees given here. On the target side, the CoCoMac database provides data from anterograde tracing studies [27, 66, 75, 76].

Missing inputs in the model, i.e., from subcortical and non-visual cortical areas, are replaced by Poissonian spike trains, whose rate *ν*_ext_ is a free, global parameter. In the original model all populations of a particular area receive the same indegree of external inputs *K*_ext_. The only exception to this rule is area TH where the absence of granular layer 4 is compensated by an increase of the external input to populations 2/3E and 5E by 20%. To elevate the firing rates in the excitatory populations in layers 5 and 6, we increase the external drive onto these populations. The possibility of a higher drive onto these populations is left open by the sparseness of the corresponding experimental data. We enhance the external Poisson drive of the 5E population, parametrized by the *K*_5E,ext_ incoming connections per target neuron (indegree), in all areas by increasing *κ* = *K*_5E,ext_/*K*_ext_. The simultaneous increase in the drive of 6E needs to be stronger, since the firing rates in population 6E of the original model (Fig 3C) are even lower than the rates of 5E (averaged across areas: 0.09 spikes/s for 5E compared to 2 ⋅ 10^−5^ spikes/s for 6E). We thus scale up *K*_6E,ext_ linearly with *κ* such that *κ* = 1.15 results in *K*_6E,ext_/*K*_ext_ = 1.5.
